# Prevalence of diabetes, impaired fasting glucose and impaired glucose tolerance in patients with thalassemia major in Iran: A meta-analysis study

**Published:** 2017

**Authors:** Milad Azami, Ali Sharifi, Siros Norozi, Akram Mansouri, Kourosh Sayehmiri

**Affiliations:** 1 Student Research Committee, Ilam University of Medical Sciences, Ilam, Iran.; 2 Department of Internal Medicine, Faculty of Medicine, Ilam University of Medical Sciences, Ilam, Iran.; 3 Department of Cardiology, Faculty of Medicine, Ilam University of Medical Sciences, Ilam, Iran.; 4 School of Nursing and Midwifery, Ahvaz Jundishapour University of Medical Science, Ahvaz, Iran; 5 Department of Biostatistics, Research Center for Prevention of Psychosocial Impairment, Ilam University of Medical Sciences, Ilam, Iran.

**Keywords:** Diabetes, Impaired Fasting Glucose, Impaired Glucose Tolerance, Thalassemia Major, Iran, Meta-Analysis

## Abstract

**Background:**

This study aimed to investigate the prevalence of diabetes, impaired fasting glucose (IFG) and impaired glucose tolerance (IGT) in Iranian patients with thalassemia major.

**Methods:**

The current study has been conducted based on PRISMA guideline. To obtain the documents, Persian and English scientific databases such as Magiran, Iranmedex, SID, Medlib, IranDoc, Scopus, PubMed, ScienceDirect, Cochrane, Web of Science, Springer, Wiley Online Library as well as Google Scholar were searched until December 2015. All steps of the study were conducted by two authors independently. To the high heterogeneity of the studies, the random effect model was used to combine studies. Data were analyzed using STATA Version 11.1 software.

**Results:**

Thirty-two studies involving 3959 major thalassemia patients with mean age of 16.83 years were included in the meta-analysis. The prevalence of diabetes in Iranian patients with thalassemia major was estimated as 9% (95% CI: 6.8-10.5) and estimated rate was 12.6% (95% CI: 6.1-19.1) for males and 10.8% (95% CI: 8.2-14.5) for females. The prevalence of IFG and IGT were 12.9% (95% CI: 7-18.8) and 9.6% (95% CI: 6.6-12.5) respectively. No relationship between serum ferritin and development of diabetes was noted.

**Conclusion:**

The prevalence of diabetes, IFG, and IGT in patients with thalassemia major in Iran is high and accordingly requires new management strategies and policies to minimize endocrine disorders in Iranian patients with thalassemia major. Screening of patients for the early diagnosis of endocrine disorders particularly diabetes, IFG, and IGT is recommended.

Thalassemia major is a hereditary hemolytic disease with a *severe form of β-*thalassemia*. *It causes severe anemia after a *reduced production of β-globin chains* (1). Thalassemia belt is expanding in the eastern *coast of the Mediterranean region*, throughout the Arabian Peninsula, Turkey, Iran, India and the Southeast Asia (2). This disease is one of the most common hereditary diseases in Iran, and the number of patients with thalassemia major is about 18800 people (3). These patients regularly receive blood to prevent complications like chronic anemia and bone changes (4). Over the past 2-3 decades, blood transfusions have significantly increased lifetime and life expectancy in patients with thalassemia major (5). At the same time, the increasing use of this treatment has led to complications of iron overload (6). One of the toxic effects of iron overload occurs in the endocrine glands (7).

Even with careful management of patients, disorders of endocrine glands such as growth retardation, hypogonadism, insulin-dependent diabetes, hypothyroidism, hyporparathyroidism may occur (8-11). To prevent this complication of iron overload, chelation therapy regimens are used (12). 

Endocrine gland complications may be due to the unsystematically iron chelation therapy in patients with thalassemia major in the developing countries (10). Diabetes is one of the most common and serious diseases that can be considered as the human’s most important metabolic disease (13). 

The most common complications of diabetes are cardiovascular diseases, retinopathy, neuropathy, nephropathy, sexual dysfunction and infection (14). The term impaired glucose tolerance (IGT) was introduced in 1979 by the National Diabetes Data Group (NDDG) as part of the classification and diagnostic criteria. Later on, this term was implemented by the World Health Organization (WHO) criteria (15). 

Progress from normal glucose to type-II diabetes has often been an intermediate state associated with change glucose metabolism called IGT or pre-diabetes stage. IGT is a risk factor for type-II diabetes and patients with IGT without lifestyle change may develop to type-II diabetes in ten years (16, 17). Whereas pre-diabetes state was detected at early stage, diabetes can be delayed for several years with appropriate iron chelation therapy and regularly use of desferal (18, 19). 

A simple review of the literature showed that the prevalence of diabetes, impaired fasting glucose (IFG) and IGT in patients with thalassemia major in Iran had been reported differently (19-22). Systematic review and meta-analysis study of the review of all literature and combining them can be a comprehensi veview of the problem in a specific population (23-25). Because of no available comprehensive report, this study assesses the prevalence of diabetes, IFG, and IGT in patients with thalassemia major in Iran.

## Methods

This review was conducted based on PRISMA (Preferred Reporting Items for Systematic Reviews and Meta-Analyses) guideline (24). To avoid bias, all steps of the study including search, selection of studies, quality assessment, and data extraction were conducted by two researchers, independently. Any disagreement was reviewed by third researcher. 

**Search Strategy:** To obtain the related documents in Persian and English, scientific databases such as Magiran, Iranmedex, SID, Medlib, IranDoc, Scopus, PubMed, Science direct, Cochrane, Web of Science, Springer, Wiley Online Library, and Google Scholar were searched until December 2015. Persian and English MeSH keywords were used. Prevalence, diabetes, Glucose Intolerance, prediabetes, endocrine disorders, ferritin, hemosiderosis, iron overload, chelation therapy, endocrine, Iran, thalassemia major and also word combination of and & or operators were used as keywords.

**Inclusion Criteria: **Related papers about the prevalence of diabetes, IFG, and IGT in thalassemia major patients in both English and Persian Language were considered as inclusion criteria. Diabetes was determined according to World Health Organization (WHO) and American Diabetes Association (ADA). The criterion for the diagnosis of IFG was determined as 100≥FBS<126 mg/dl while the criterion for IGT was determined as two-hour glucose levels of 7.8-11.1 mmol/L(140-200 mg/dl) on the 75 g oral glucose tolerance test (26).

**Exclusion Criteria:** Studies with non-randomly selected sample size; lack of relevance to the topic; letters to the editor and case report studies.

**Evaluation of Quality: **Researchers using a *STROBE* *standard checklist *(27) including 22 items. The selected studies were appraised in all aspects of methodology including sampling methods, measurement parameters, statistical analysis and objectives of the study. The minimum and maximum scores in this checklist were 16 and 44, respectively. The papers that had reached the minimum score (16) were selected for the meta-analysis stage. 

**Figure1:**
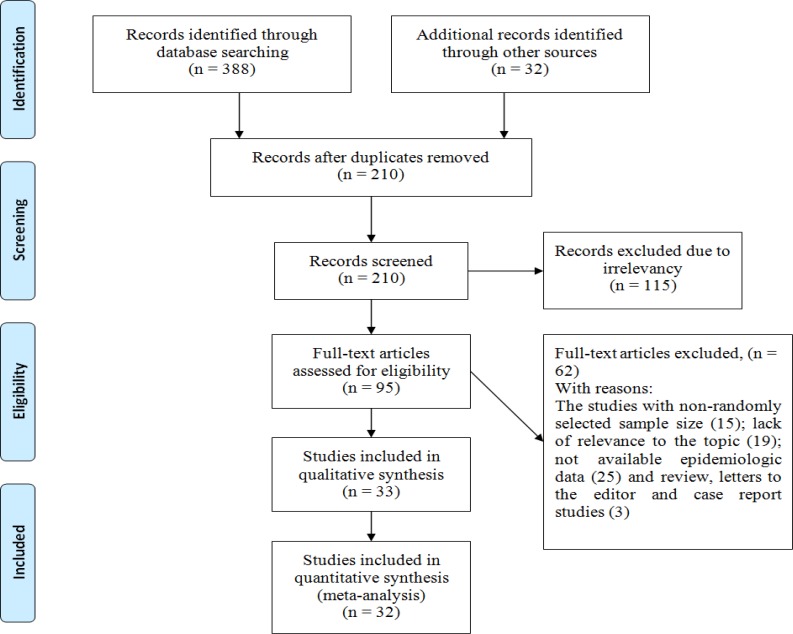
The entrance steps of systematic review and meta-analysis

**Study Selection: **In the initial search, 420 studies probably related to the prevalence of diabetes, IFG, and IGT in patients with thalassemia major were found of which 210 studies were excluded because they were duplicate papers (papers extracted by two researchers with identical titles, authors, and journal). From the remaining 210 studies, 189 cases were excluded after reading the summary and the full text of the paper due to non-relevance of the topic, and the lack of criteria and low quality (figure 1).

**Data Extraction: **All final papers imported to study process were extracted by a pre-prepared checklist. The checklist includes the author name, year of study, place of study, type of study, sample size, prevalence of diabetes, IFG and IGT, prevalence of diabetes, IFG and IGT according to gender, *diagnostic criteria for *diabetes, IFG and IGT and mean of serum ferritin level in diabetes and control groups.

**Statistical Analysis: **The variance of each study was calculated according to the binomial distribution. According to the sample size and variance, the studies were combined. To assess the heterogeneity of the studies, Cochran test, and I^2^ index was used. The heterogeneity of the study was 84.8% classified among studies with high heterogeneity (I^2^ index less than 25%: low heterogeneity, 25%-75%: average heterogeneity and more than 75%: high heterogeneity). Due to the heterogeneity of the studies, the random effects model was used to combine studies. To find the source of heterogeneity among studies, meta-regression model was used for the year of study, sample size, quality of studies and diagnostic method. To investigate propagation bias, Beggs test and draw of funnel plot were used. Data were analyzed using the Stata Version 11.1 software. The significance level was considered as p<0.05.

## Results

In a systematic review of studies, 32 studies were included into the meta-analysis process. All participants in the study were 3959 with thalassemia patients in an average age of 16.83 years (95% CI: 15.71-17.94) (table 1).

**Table 1:** Detailed characteristics of 32 articles included in the systematic review on the prevalence of diabetes, IFG and IGT in patients with thalassemia major

**Ref.**	**Author Name**	**Place of study**	**Year of study**	**Sample size**	**Age** **(Mean±SD)**	**Diagnostic criteria for diabetes**	**Prevalence of Diabetes** ** (%)**	**Prevalence of ** **IGT (%)**	**Prevalence of ** **IFG (%)**
^19^	Najafipour	Tabriz	2006	65	15.6±4.4	DAD and WHO	8.9	7.1	28.8
^20^	Rostami	Bushehr	2009	60	20.23±23	WHO	18.3	6.7	
^21^	Safari	Qazvin	2006	63	20.89±5.01	DAD	25.4		
^22^	Rezaei	Kohgiluyeh va boyer ahmad	2003	233	13.24±6.1	DAD	3.1		4
^28^	Kashanchi Langarod	Karaj	2010	184	19.64±7.06	DAD	10.22		12.4
^29^	Najafipour	Tabriz	2005	56	15.6±4.25	DAD and WHO	8.9	7.1	30
^30^	Company	Ahwaz	2003	195	14.9±6.09	DAD and WHO	16.4	19	
^31^	Kawsarian	Sari	1996	70		DAD and WHO	28.5	15.7	1
^32^	Mortazavi	Zanjan	1991	146		DAD and WHO	2.7	6.2	
^33^	Soheili Kha	Yazd	1998	53	10.7	DAD	3.7		
^34^	Keihanian	Tehran	2010	133	18.28	DAD	6		
^35^	Azimi	Tehran	2003	45		DAD and WHO	11.1		
^36^	Mahdavi Anari	Tehran	1999	60		DAD	8.3		
^37^	Fotoohi	Tehran	1999	60		DAD and WHO	18.3	8.3	
^38^	Younesi	Qazvin	1998	94		DAD	5.3		
^39^	Yazdi	Yazd	2005	65	10.3	DAD	8.3	18.3	
^40^	Haghverdi	Sari	1998	20		DAD	5		20
^41^	Shiva	Tabriz	2006	71	12.9±5.2	DAD and WHO	8.5	21.1	
^42^	Ishraqi	Babol	2010	280	19.6±8.5	DAD and WHO	13.9		
^43^	Jahantigh	Zahedan	2011	346	17.7±4.9	DAD and WHO	15.9	6.6	
^44^	Faalpur	Ardebil	2002	51		WHO	3.9	3.9	
^45^	Saffari	Qazvin	2012	77	21.26±4.53	WHO	16.9	13	
^46^	Arjmandi Rafsanjan	Tehran	2004	273		DAD	18.3		
^47^	Eshghi	Zahedan	2001	66		DAD and WHO	4.5	3	
^48^	Moayeri	Tehran	2006	158	4.8	DAD	10.1		
^49^	Karamifar	Shiraz	2003	150		DAD	7.3		
^50^	Mowla	Sari	2004	98	4	DAD	8.2		
^51^	Shams	Tehran	2009	78	16±6	DAD	5.1		12.8
^52^	Karimi	Shiraz	2008	47	19.7±5.3	DAD and WHO	19		
^53^	Raissi	Shahrekord	2003	40		DAD and WHO	10		
^54^	Sadat	Gorgan	2007	185		DAD	11.9		
^55^	Mehrvar	Tehran	2004	437		DAD	6.2		

**Figure 2:**
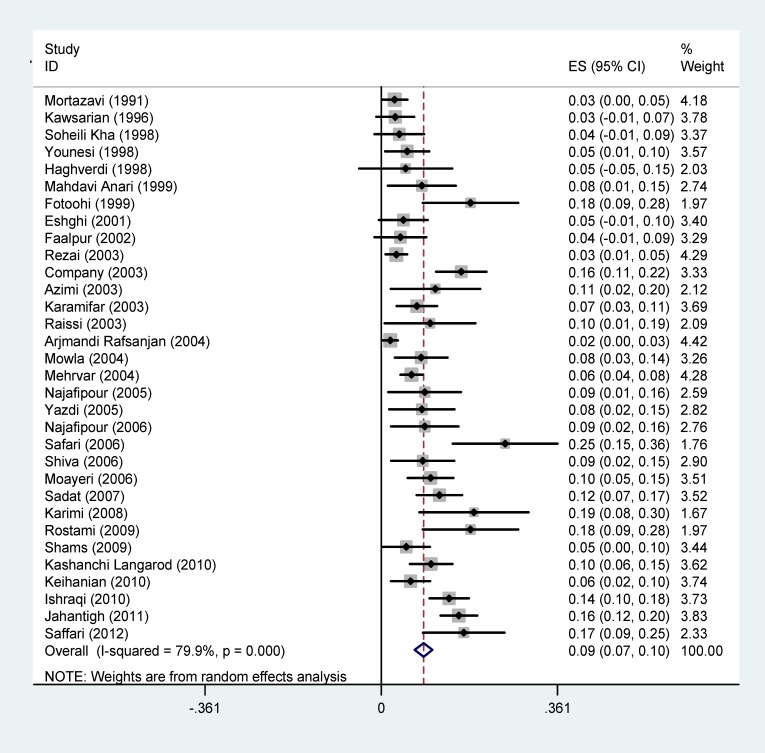
Forest plots presenting the prevalence of diabetes in patients with thalassemia major. Weights are assessed from the random-effects model analysis

**Diabetes: **The prevalence of diabetes in patients with thalassemia major in Iran was estimated as 9% (95% CI: 6.8-10.5). The lowest prevalence of diabetes is associated with *Arjmandi's *(2004) in Tehran (1.8%) and the highest prevalence of diabetes was reported in Safari’s (2006) in Qazvin (25.4%) (figure 2).

The prevalence of diabetes in patients with thalassemia major had been examined in 7 studies according to sex. This rate was estimated as 12.6% (95% CI: 6.1-19.1) in males and 10.8% (95% CI: 8.2-14.5) in females. 

**Figure 3:**
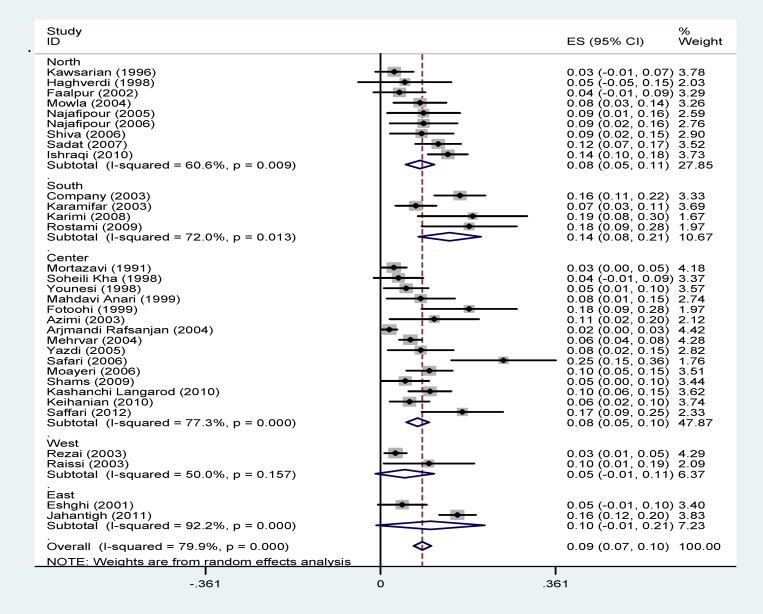
Forest plots presenting the prevalence of diabetes in patients with thalassemia major sub-grouped by geographical regions.

The prevalence of diabetes in patients with thalassemia major was indicated according to the geographical regions in figure 3 and shows that the lowest prevalence was in the West of Iran (5%) in which the highest prevalence rate was in South (14.3%). 

In terms of the diagnostic criteria for diabetes, the lowest and the highest prevalence rates are related to *DAD* (7%) and the history of insulin therapy (13%) (table 2). 

**Table 2:** Estimates for prevalence of diabetes in patients with thalassemia major according to diagnostic criteria for diabetes

**Diagnostic criteria**	**(N) studies**	**Sample size**	**I** ^2 ^ **(%)**	**Confidence interval (%)**	**Overall prevalence (%)**
DAD	16	2284	73.3	5-9	7
WHO	3	188	80.7	2-13	12
WHO And DAD	13	1487	82.7	6-13	10

**Figure 4:**
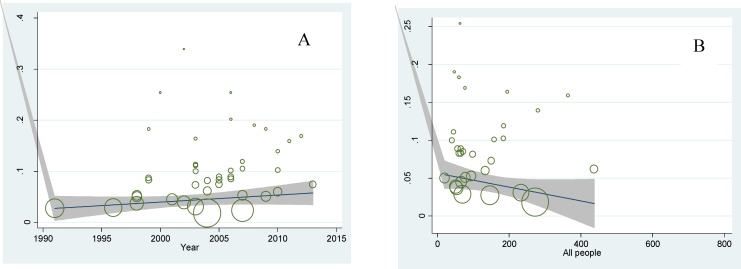
A: Meta-regression plot of the prevalence of diabetes based on the year of study (P=0.382). B: Meta-regression plot of the prevalence of diabetes based on sample size of the study (P=0.362

Regression model was used to investigate the relationship between prevalence of diabetes with the year of study and sample size. The p-values were calculated 0.382 and 0.326, respectively and no statistically significant was found (figure 4). 

**Figure 5:**
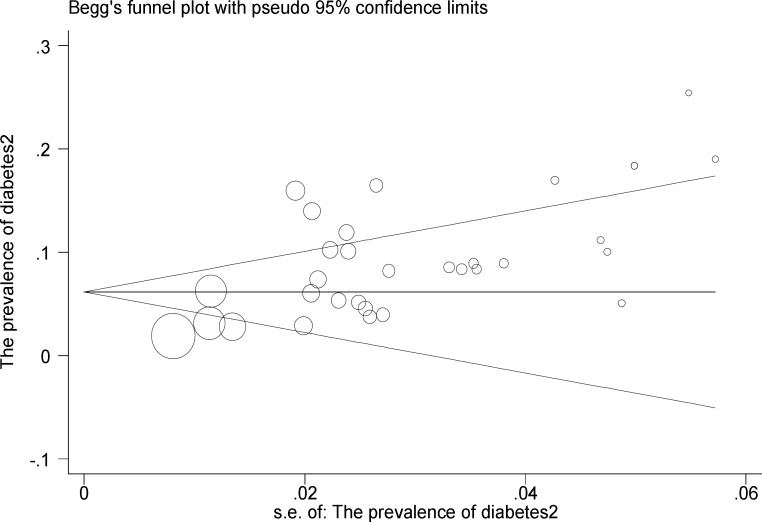
Publication bias for the prevalence of diabetes in patients with thalassemia major (P=0.435). The size of circles shows the weight of studies (bigger circles represent more samples

**Figure 6:**
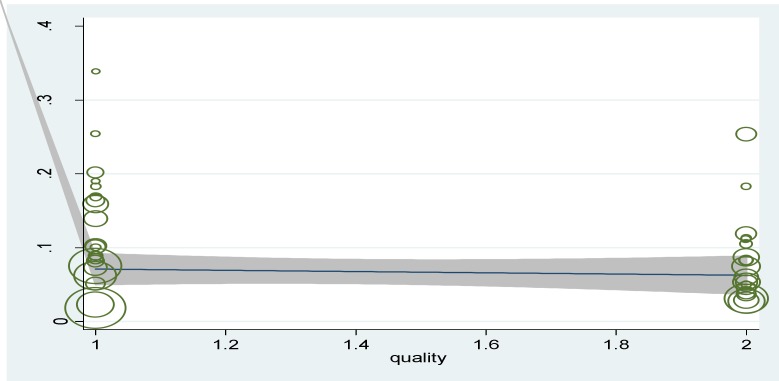
Meta-regression plot of the prevalence of diabetes based on the quality of the study (P=0.187

**Figure 7:**
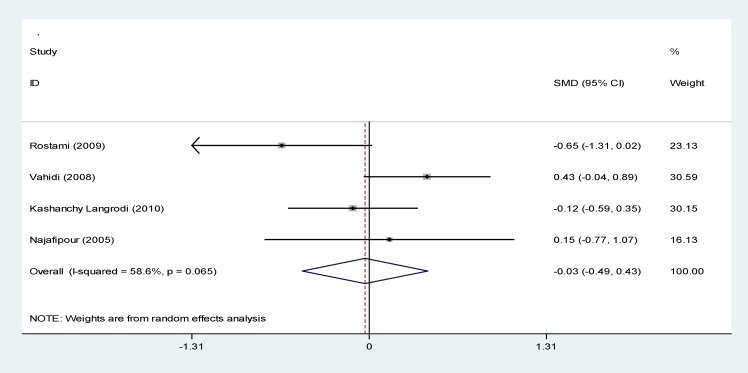
Forest plots presenting the relationship between serum ferritin level and diabetes based on a random effects model in the meta-analysis. SMD indicates the standardized mean difference

In figure 5 publication bias is shown as symmetry in a funnel plot and bias is not involved in these studies (P=0.345). In figure 6 the relationship between prevalence of diabetes with studies of quality was provided and p-values were calculated 0.187 and no significant relationship was observed in this regard. In 4 studies investigated, between serum ferritin levels and diabetes, no significant relationship was found (p<0.05) (figure 7).

**Figure 8:**
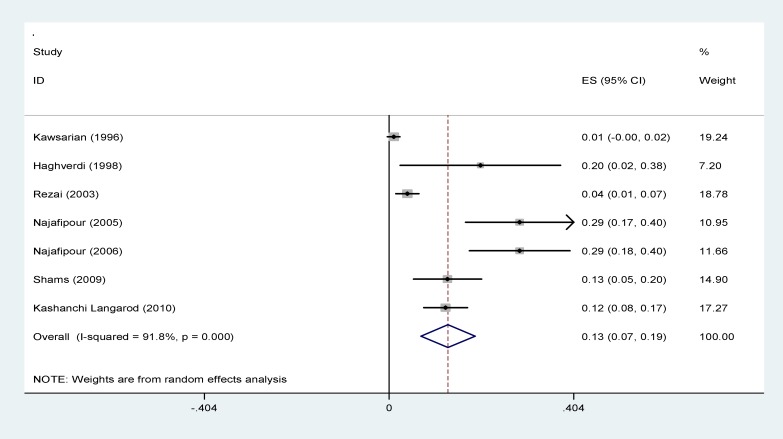
Forest plots presenting the prevalence of impaired fasting glucose in patients with thalassemia major

**IFG: **The prevalence of IFG in patients with thalassemia major in Iran was shown in figure 8 and this rate was estimated as 12.9% (95% CI: 7-18.8).

**Figure 9:**
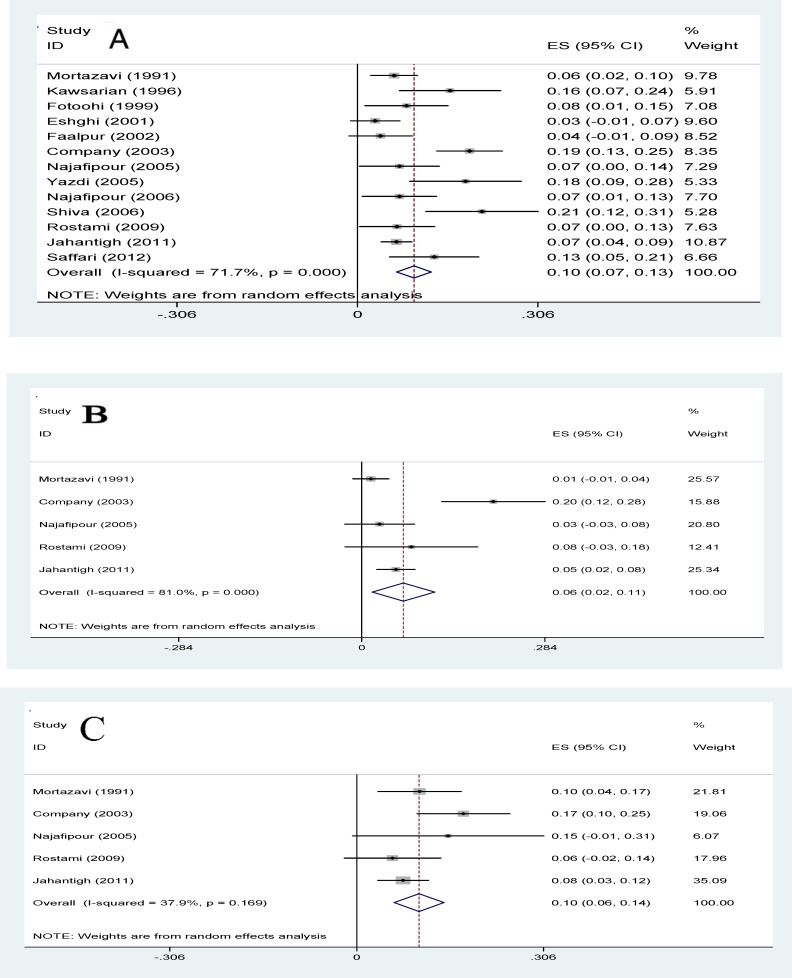
Forest plots presenting the prevalence of impaired glucose tolerance in total (A) male (B) and female (C) patients with thalassemia major

**Figure 10:**
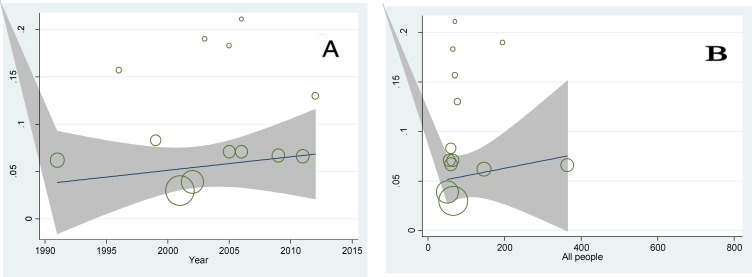
A: Meta-regression plot of the prevalence of IGT based on the year of study (P=0.702). B: Meta-regression plot of the prevalence of IGT based on sample size of the study (P=0.736

**IGT: **In 13 studies, the prevalence of IGT in patients with thalassemia major in Iran was estimated 9.6% (95% CI: 6.6-12.5). The lowest prevalence of IGT was related to a study in 2001 in Zahedan (3%) and the highest prevalence of IGT was related to a study in 2006 in Tabriz (21.1%). In 5 studies, the prevalence of IGT in patients with thalassemia major had been investigated according to sex estimated in males & females as 6.5% (CI 95%: 1.6-11.3) and 10.2% (CI 95%: 6.1-14.3), respectively (figure 9). The relationship between IGT in patients with thalassemia major with the year of study and sample size, meta-regression model was used and the p-values were calculated 0.702 and 0.736, respectively and no significant correlation was found (figure 10).

## Discussion

The overall prevalence of diabetes in patients with thalassemia major was estimated 9 percent, the prevalence of diabetes in patients with thalassemia major in other countries was reported 6-27%, (Such as: United Arab Emirates (10.5%), Oman (27%), Taiwan (26.8%), South America (14%) and Italy (6.5%) (56-60). Genetic, geographical, cultural and economic factors as well as the quality of blood transfusion and chelation therapy, especially onset and the rate of the desferal dosage can be causes of different in reporting prevalence in various countries. . A systematic review of Iranian thalassemia patients has reported the regular iron chelation therapy as 54%. Therefore, chelation therapy in many patients with thalassemia major in Iran has been done unsystematically that must be considered (61).

The difference in reporting the prevalence of diabetes in patients with thalassemia major in Iran was 1.8-34% and it seems the most prospective reason for this difference, different diagnostic criteria. Therefore, the subgroup analysis was performed on diagnostic criteria and most studies (16 studies) had used ADA criteria, and this rate was estimated 7% and was not significantly different. Also, in the results of meta-regression model, no significant difference was found based on diagnostic criteria (P=0.343).

In a systematic review study in Iran, the prevalence of diabetes among adults is 3% and 16.8%, respectively for the age range of 25-34 and 55-64-years-old (62). In another review study in Iran, the prevalence of diabetes among the 15-25 years-old (young society) has been reported about 3.6% (63).  And both studies indicated the incidence of diabetes *significantly increases with age*. In the present study, the age range of patients was 10-20 years-old (mean age of 16.8), which the prevalence of diabetes was estimated 9% that is more than the non-thalassemic population with same age. In some studies, the obvious role of iron overload has been proven in the endocrine glands including pancreas in the pathogenesis of diabetes (64,65) and other studies have shown that insulin resistance and lack of insulin are the two reasons of pre-diabetes and diabetes in this patients (66, 67). Endocrine complications in patients with thalassemia major mostly happen in their second decade of life. The highest prevalence of diabetes is reported in Razavi (33.9%) (22), Saffari (25.4%) (21) and Rabbani’s (25.4%) (32), and the lowest prevalence occurred in Arjmandi (1.8%) (41) and Mortazavi (2.7%) (27) and the results were not highly different in terms of average age of subjects participating in studies but the prevalence of diabetes was variable that can indicate different therapeutic follow-ups of these patients in different parts of Iran. The most comprehensive study in terms of sample size and examination areas in Iran was in Mehrvar’s et al. (50) in 2004 with a sample size of 407 thalassemia patients in Shiraz was reported a prevalence of diabetes as 6.6% which was consistent with the present results. The prevalence of diabetes in male patients with thalassemia major (12.6%) is more than female patients (10.8%), but this difference was not significant. A review study in the general population of Iran has estimated the prevalence of diabetes in males and females, 1.7% and 3.8%, respectively (62), which was inconsistent with results of this study. The most obvious reason can be the role of iron overload in thalassemia patients that its pathogenesis has been proven in endocrine disorders (7).

The prevalence of diabetes in patients with thalassemia major in studies of high and moderate quality was estimated as 9% and 11%, respectively and results of, no significant correlation was found between the prevalence of diabetes and quality of studies (P=0.187) in meta-regression model, which poor-quality studies can be the cause of that.

In the present study, in the relationship between serum ferritin level and diabetes, a mean difference of serum ferritin in case and control groups was estimated as -0.3 (95% CI:-49 to 43) and was not a significant. In other studies, different results were reported, in Mula-Abed’s et al. (57) there was not a significant relationship but in Borgna’s (64) and Gamberini’s studies (65) there was a significant relationship. The prevalence of IFG in patients with thalassemia major in Iran was estimated to be 9.6%. In a review study, the prevalence of IFG in the adult population of Iran has been reported as 16.8% (62) and it also showed that with increasing age, the prevalence was increased. Due to the small number of studies, could not do a sub-group analysis on the prevalence of IFG.

The overall prevalence of IGT in Iranian patients with thalassemia major was estimated as 9.6%. Due to the low number of studies, we could not sub-group based on the IGT. The prevalence of this disorder in other countries including Turkey (2.2%), Italy (6.5%), Thailand (12.5%) and Egypt (24.1%) was variable (68-71). 

The prevalence of IGT in patients with thalassemia major in 5 studies under review on females (10.2%) was more than males (6.5%). However, this difference was not significant.

Meta-regression model for finding the source of heterogeneity among studies was used, and meta-regression results in a year of studies and sample size for diabetes and IGT prevalence was not statistically significant. During years of studies (1991-2015), the prevalence of diabetes and IGT has been almost constant. Constant prevalence of the diseases over the last 24 years, and also the high prevalence of diabetes in patients with thalassemia major, attention, and follow-up in this patient seems necessary.

No relationship between serum ferritin and development of diabetes was noted. Jiang et al. was found a strong relationship between ferritin and diabetes (72), also high ferritin level is associated with cardiovascular disease, hepatic steatohepatitis and central adiposity (72-74). 

Publication bias for studies entering the meta-analysis process has been shown as symmetry in Funnel plot in which the p-value was 0.345, indicating that the possibility of publication bias is not statistically significant. 

Research limitations**: **1. The inability of internal databases for searching the combined keywords. Thus, we cannot use the keywords in combination; 2. Because of no the prevalence of diabetes, IFG, and IGT by age reported in studies, we could not calculate the prevalence based on age; 3. Since desferal dosage and intervals of blood transfusion were not reported in most studies, we could not calculate the relationship between these variables with diabetes, IFG and IGT; and 4. Due to the limited number of studies, we could not do a subgroup analysis of studies on the prevalence of IFG and IGT.

In conclusion the prevalence rates of diabetes, IFG, and IGT are high in Iranian patients with thalassemia major. Therefore, more effective protocols and management strategies which include improved protocols, blood transfusion, chelation therapy, educating and enhancing awareness of the parents and patients about iron overload complications seem to be essential to minimize endocrine complications. In addition, screening for the early diagnosis of endocrine complications once every six-month should be done as suggested by the Thalassemia International Federation. 
